# Influence of shortened recovery between resistance exercise sessions on muscle‐hypertrophic effect in rat skeletal muscle

**DOI:** 10.14814/phy2.14155

**Published:** 2019-06-28

**Authors:** Junya Takegaki, Riki Ogasawara, Takaya Kotani, Yuki Tamura, Ryo Takagi, Koichi Nakazato, Naokata Ishii

**Affiliations:** ^1^ Department of Life Sciences, Graduate School of Arts and Sciences The University of Tokyo Tokyo Japan; ^2^ Ritsumeikan Global Innovation Research Organization Ritsumeikan University Kusatsu Shiga Japan; ^3^ Department of Life Science and Applied Chemistry Nagoya Institute of Technology Nagoya Japan; ^4^ Graduate School of Health and Sport Science Nippon Sport Science University Tokyo Japan

**Keywords:** Inflammation, mitochondria, mTOR, proteolysis

## Abstract

Resistance exercise training induces muscle hypertrophy, and recovery between sessions is one of the major determinants of this effect. However, the effect of the recovery period between sessions on muscle hypertrophy following resistance exercise training remains unclear. To elucidate the effect of recovery period on hypertrophy, in the present study, we investigated changes in protein degradation systems and hypertrophic responses in rat skeletal muscle to resistance training with variable recovery periods. In the conventional recovery group (exercised every 72 h) and a shorter recovery group (exercised every 24 h), 18 bouts of resistance exercise consisting of 50 repetitions of a 3‐sec maximal isometric contraction caused muscle hypertrophy and slight activation of muscle protein degradation systems. By contrast, in an excessively shorter recovery group (exercised every 8 h), 18 bouts of resistance exercise did not cause hypertrophy and markedly activated protein degradation systems, accompanied by inflammatory responses. These observations indicate that excessive shortening of recovery between sessions does not cause skeletal muscle hypertrophy, likely due to the activation of proteolysis induced by inflammatory responses to resistance exercise training.

## Introduction

Resistance exercise training is widely recognized as an effective tool for increasing skeletal muscle mass and strength. Recovery between bouts is one of the major determinants of the exercise training effect, and excessively short recovery periods between repetitions can lead to insufficient acute responses (Coffey et al., 2007; Takegaki et al., 2017). However, the influence of a shortened recovery period on the muscle‐hypertrophic effects of multiple sessions of resistance exercise and the mechanisms involved in that are unclear.

Skeletal muscle mass is regulated by the net balance between protein synthesis and degradation, with a balance positive resulting in muscle hypertrophy (Phillips et al., 1997). Resistance exercise can activate either of these responses. In skeletal muscle, two proteolytic systems are known to mostly account for protein degradation: the ubiquitin‐proteasome system and the autophagy‐lysosome system. In the ubiquitin‐proteasome system, damaged proteins are targeted by ubiquitination, and degradation is catalyzed by the 26S proteasome. It is well known that contractile proteins (e.g., myosin heavy and light chains, actin) are mainly degraded by this system (Clarke et al., 2007; Cohen et al., 2009; Polge et al., 2011). Additionally, a previous study demonstrated that four repetitions of resistance exercise with a short recovery (3 h) acutely activate the mRNA expression of atrogin‐1, a muscle‐specific ubiquitin ligase that activates protein ubiquitination (Coffey et al., 2007). Therefore, it has been proposed that the ubiquitin‐proteasome system is involved in adaptive changes related to the shortening of recovery in resistance exercise training. In the autophagy‐lysosome system, cellular components are enveloped in an isolation membrane and formed into an autophagosome. These autophagosomes are then fused with lysosomes and degraded (Green and Levine, 2014). Previous studies have reported that resistance exercise training does not alter autophagosome formation {indicated by Microtubule‐associated protein 1 light chain 3 (LC3) ‐II} in rat skeletal muscle (Ogasawara et al., 2016; Kwon et al., 2018). However, the effect of shortening the recovery period in between multiple sessions of resistance exercise on these systems has not been elucidated.

Recently, growing evidence has demonstrated that resistance training improves not only skeletal muscle mass but also mitochondrial function in skeletal muscle (Salvadego et al., 2013; Porter et al., 2015). Mitochondrial dynamics (fusion and fission) are thought to play important roles in the function of mitochondria, and some previous studies have reported that resistance training affects these dynamics in skeletal muscle (Kitaoka et al., 2015, 2016a, 2016b). Moreover, factors related to mitochondrial function and dynamics may alter protein proteolytic systems. Tezze et al. reported that loss of Optic atrophy protein 1 (OPA1), a factor regulating mitochondrial fusion, activates the ubiquitin‐proteasome system and autophagy, resulting in muscle atrophy in mice (Tezze et al., 2017). Romanello et al. reported that expression of mitochondrial fission 1 protein (FIS1) and dynamin‐related protein 1 (DRP1), factors regulating mitochondrial fission, promotes skeletal muscle atrophy in mice (Romanello et al., 2010). Additionally, others and we have reported that exhaustive resistance exercise causes oxidative stress in skeletal muscle (Haraguchi et al., 2011; Takegaki et al., 2017), and this stress is known to exert a toxic effect on mitochondria, resulting in mitochondrial fragmentation and dysfunction (Fan et al., 2010; Iqbal and Hood 2014). Therefore, excessive shortening of the recovery period between sessions may lead to mitochondrial dysfunction and the activation of skeletal muscle degradation systems during resistance training.

As another possibility, several factors are known to be activated in response to the repetition of resistance exercise with short recovery periods. Previous studies have reported that the repetition of resistance exercise with a short recovery period results in the phosphorylation of 5′ AMP‐activated protein kinase (AMPK) and activates inflammatory signals (Coffey et al., 2007; Takegaki et al., 2017). AMPK is a key sensor of energy status in skeletal muscle and an activator of the ubiquitin‐proteasome system and autophagy‐lysosome system (Nakashima and Yakabe, 2007; Mao and Klionsky, 2011; Sanchez et al., 2012). Additionally, pro‐inflammatory cytokines {e.g., tumor necrosis factor‐*α* (TNF‐*α*), interleukin (IL)‐1*β*, IL‐6} are also known to activate these systems (Bodine et al., 2001a, 2001b; Cai et al., 2004; Janssen et al., 2005; Doyle et al., 2011). However, while these factors are known to be activated after a few sessions of resistance exercise with short recovery, the changes in the response to multiple sessions of exercise and the influence on muscle‐hypertrophic effects are unclear.

As stated above, the effect of shortening recovery between bouts on protein degradation systems has been partially reported, but the detailed changes in multiple bouts causing muscle hypertrophy have not been elucidated. In addition, how the hypertrophic effect changes and the mechanisms underlying the alteration of the activation of protein degradation systems are also not fully understood. Therefore, in the present study, we investigated changes in muscle‐hypertrophic effects and proteolytic systems including the associated mechanisms related to shortened recovery periods between 18 bouts of resistance exercise. We hypothesized that excessive shortening of the recovery period would attenuate the muscle‐hypertrophic effect by upregulating the proteolytic systems during repeated resistance exercise.

## Materials and Methods

### Animals and experimental design

Twenty‐one male Sprague‐Dawley rats (10 weeks old) were obtained from CLEA Japan (Tokyo, Japan). All animals were housed in an environment maintained at 22 ± 2°C under a 12‐h/12‐h light–dark cycle and were provided with food and water ad libitum. Rats were randomly divided into three groups, which were subjected to resistance exercise every 72 (*n* = 7), 24 (*n* = 7), and 8 h (*n* = 7). In order to match the ages at the endpoint, the 72‐h, 24‐h, and 8‐h groups began exercising at 11, 15, and 17 weeks of age, respectively. All animals completed 18 sessions of resistance exercise. Forty‐eight hours after the last exercise session, rats were anesthetized and exsanguinated, and muscle samples were collected. Before dissection, rats were fasted overnight. Collected muscles were frozen at –80°C until use. This study was approved by the Ethics Committee for Animal Experiments at Nippon Sport Science University.

### Resistance exercise protocol

Resistance exercise was carried out according to the protocol described by Ogasawara et al. (2014). Briefly, under isoflurane anesthesia, the hair on the right hind limb of each rat was shaved, and the shaved portion was cleaned with alcohol wipes. The right foot of each rat was firmly attached to the foot plate (the ankle joint angle was positioned at 90°) in the prone position, and the gastrocnemius muscle was stimulated percutaneously with electrodes (Vitrode V, Ag/AgCl; Nihon Kohden, Tokyo, Japan). Resistance exercise was performed by maximal isometric contractions (3 sec × 10 contractions with a 7‐sec interval between contractions, for five sets with 3‐min rest intervals). The voltage (~30 V) and stimulation frequency (100 Hz) were adjusted to produce maximum isometric contraction. The left gastrocnemius muscle served as an internal control. A preceding study reported that 12 sessions of resistance exercise (three sessions per week) using this method induced muscle hypertrophy (Ogasawara et al., 2016). Torque signals were collected continuously at a sampling rate of 2000 Hz, with a 16‐bit analog‐to‐digital converter (PowerLab/16SP; AD Instruments, Sydney, Australia) and analyzed with Power Lab Chart 5 software (AD Instruments). Total impulse was also analyzed by integrating the torque value with time using the software.

### Muscle cross‐sectional area

Gastrocnemius cryosections (8 *μ*m) were fixed with 4% paraformaldehyde for 15 min and blocked with 5% goat serum for 60 min at room temperature. Following blocking, anti‐laminin primary antibody (L8271, Sigma‐Aldrich, St. Louis, MO) in phosphate‐buffered saline (PBS) containing 3% goat serum was applied overnight at 4°C. Sections were then incubated for 1 h with goat anti‐mouse fluorescent‐conjugated secondary antibody at room temperature. According to the quantification procedure described in a previous study (Ogasawara et al., 2016), the cross‐sectional areas (CSAs) of approximately 300 randomly selected myofibers per muscle were measured with ImageJ software (National Institutes of Health, Bethesda, MD).

### Western blotting

For western blotting analysis, muscle samples were homogenized and analyzed as described previously (Takegaki et al., 2017). Briefly, muscle samples were homogenized in RIPA buffer (Thermo Fisher Scientific, Waltham, MA) containing Halt^TM^ protease and phosphatase inhibitor cocktail (Thermo Fisher Scientific) and were centrifuged at 10,000*g* for 10 min at 4°C. After determination of supernatant concentrations, samples were diluted in 3× Blue Loading Buffer (Cell Signaling Technology, Danvers, MA) and boiled at 95°C for 5 min. Equal amount of proteins (20 *μ*g) were then subjected to 7.5%, 10%, or 12% TGX gel (BioRad, Hercules, CA) and subsequently transferred to Polyvinyliden difluoride (PVDF) membranes. Membranes were blocked in 5% skim milk in Tris‐buffered saline with Tween 20 (TBST) for 1 h at room temperature and subsequently incubated with the primary antibodies overnight at 4°C. Membranes were then incubated for 1 h with the appropriate secondary antibodies at room temperature and visualized using chemiluminescent reagents (Clarity^TM^ Western ECL Substrate, BioRad). Bands were detected and quantified with ChemiDoc XRS (170‐8071, Bio‐Rad).

### Primary antibodies for western blotting

The following primary antibodies were used: Ubiquitin (#3933; Cell Signaling Technology (CST) Japan, Tokyo, Japan), p‐Forkhead box O (FOXO) 1^Ser256^ (#84192; CST), FOXO1 (#2880; CST), p‐FOXO3a^Ser253^ (#9466; CST), FOXO3a (#2497; CST), LC3 (#2775; CST), p62/SQSTM (PM045, Medical & Biological Laboratory, Nagoya, Japan), p‐UNC‐551‐like kinase 1 (ULK1)^Ser757^ (#14202; CST), p‐ULK1^Ser555^ (#5869; CST), ULK1 (#8054; CST), p‐Akt^Ser473^ (#9271; CST), Akt (#4685; CST), p‐p70S6K^Thr389^ (#9205; CST), p70S6K (#2708; CST), p‐rpS6^Ser240/244^ (#2215; CST), rpS6 (#2217; CST), p‐4E‐binding protein 1 (4E‐BP1) ^Thr37/46^ (#9459; CST), 4E‐BP1 (#9452; CST), Oxidative phosphorylation (OXPHOS, ab110413; Abcam, Cambridge, UK), Peroxisome proliferator‐activated receptor‐γ coactivator‐1*α* (PGC‐1*α*, 516557; Millipore, CA), Parkin (ab77924; Abcam), OPA1 (#612606; BD Transduction Laboratories, Tokyo, Japan), Mitofusion‐2 (MFN2, ab124773; Abcam), FIS1 (ab96764; Abcam), DRP1 (ab56788; Abcam), p‐AMPK^Thr172^ (#2531; CST), AMPK (#2532; CST), 4‐hydroxy‐2‐nonenal (4‐HNE, ab8506; Abcam), p‐Tuberous sclerosis complex 2 (TSC2)^Ser1387^ (#5584; CST), TSC2 (#4308; CST), and Nuclear factor‐kappa B(NF‐*κ*B, #8242; CST).

### RNA extraction and real‐time qPCR

Total RNA was extracted from muscle samples with TRIzol (Invitrogen, Carlsbad, CA) according to the manufacturer’s instructions. RNA concentrations were measured using a NanoDrop 1000 (Thermo Fisher Scientific), and 1.5 *μ*g of total RNA was reverse‐transcribed into cDNA with a High Capacity cDNA RT kit (Applied Biosystems, Foster City, CA). Gene expression levels were quantified using qPCR reagent (THUNDERBIRD SYBR qPCR Mix, Toyobo, Osaka, Japan) and a thermal cycler with an optical reaction module (CFX96 Touch, BioRad). *GAPDH* was used as a control housekeeping gene. The sequences of the primers used in this study are shown in Table 1. Gene expression was quantified using the calibration curve method.

**Table 1:** Primer sequences for qPCR.

Gene	Forward primer (5′‐3′)	Reverse primer (5′‐3′)
*Atrogin‐1*	AAGGAGCGCCATGGATACTG	AGCTCCAACAGCCTTACTACG
*Murf‐1*	GACATCTTCCAGGCTGCCAA	TGCCGGTCCATGATCACTTC
*Il‐1beta*	AAATGCCTCGTGCTGTCTGA	TTGGGATCCACACTCTCCAG
*Il‐6*	GCAAGAGACTTCCAGCCAGT	TTGCCATTGCACAACTCTTTTCT
*Gapdh*	GTCGTGGAGTCTACTGGCGTCTT	CAGTCTTCTGAGTGGCAGTGATGG

### Quantification of Ribosomal RNA (rRNA)

For rRNA quantification, 5 *μ*L of total RNA solution (25 *μ*g muscle/*μ*L Tris‐EDTA buffer) was mixed with 1 *μ*L GR Red loading buffer (Biocraft, Tokyo, Japan) and 1 *μ*L glycerol, and loaded onto a 1% agarose gel in Tris‐acetate with EDTA buffer. The bands were detected and quantified with ChemiDoc XRS (170‐8071, BioRad).

### Statistical analysis

Data were analyzed using repeated measures two‐way ANOVA (session × recovery) for mechanical parameters and two‐way ANOVA (exercise × recovery) for other analyses. If an interaction was observed, Bonferroni multiple‐comparison testing was performed. All values are expressed as mean ± SEM. Statistical significance was indicated by *P* < 0.05.

## Results

### Maximal isometric torque, impulse, and skeletal muscle mass

**Figure 1:**
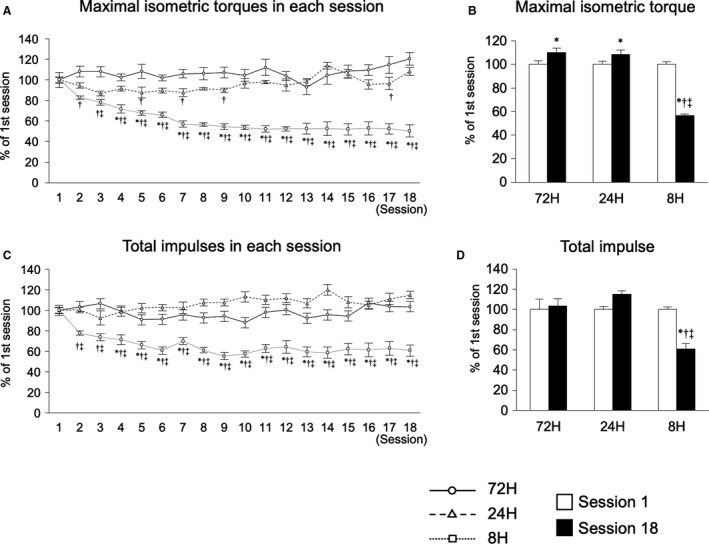
Session‐dependent changes in maximal isometric torques and total impulses in exercised muscles. Maximal isometric torques in each session (A), maximal isometric torques in first and final sessions (B), total impulses in each session (C), total impulses in first and final sessions (D). Data are expressed relative to each first session and presented as the mean + SE. *P < 0.05 versus Session 1 in each group, ^†^ *P* < 0.05 versus 72‐h recovery group, ^‡^ *P* < 0.05 versus 24‐h recovery group.

**Figure 2:**
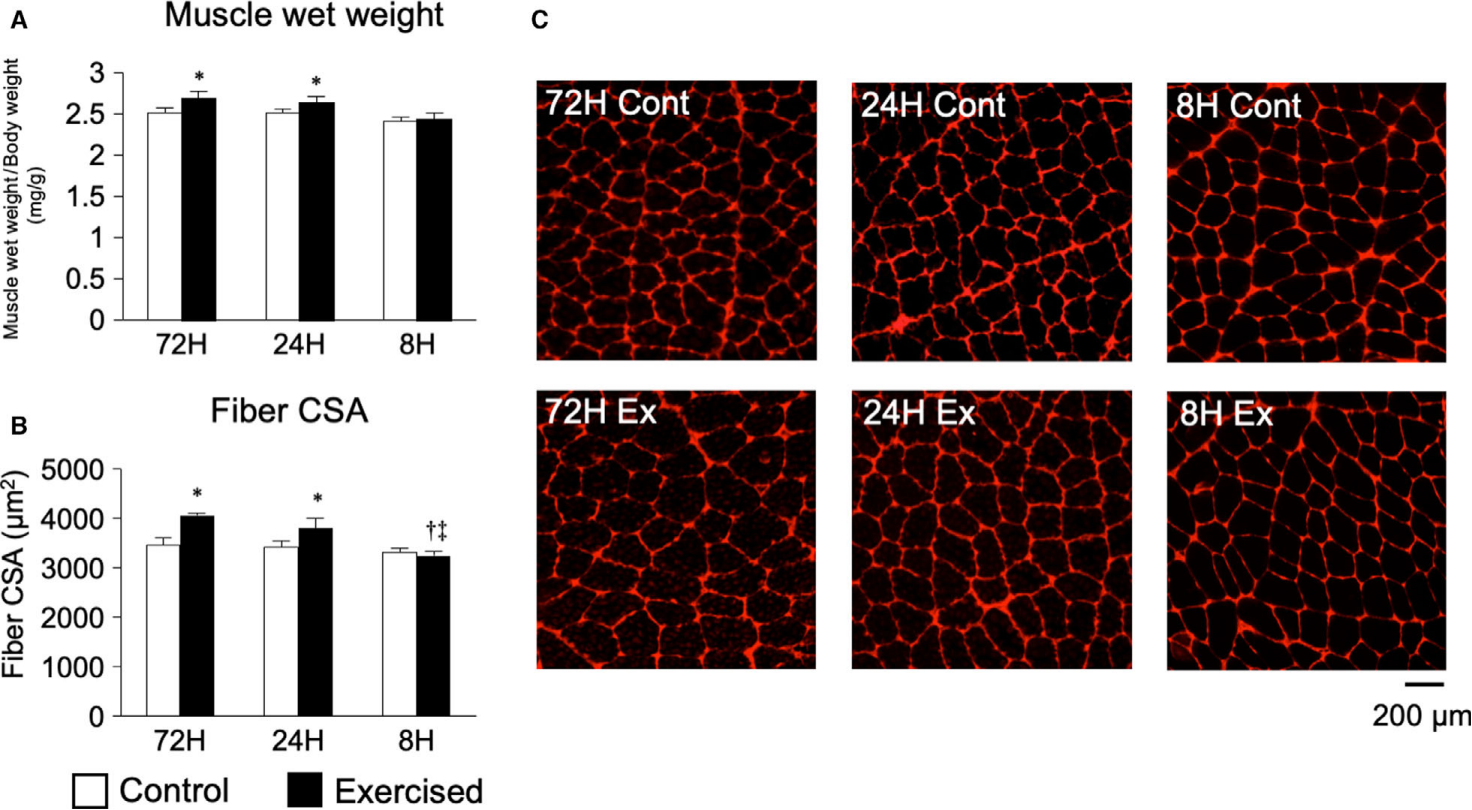
Changes in muscle‐hypertrophic effects according to the duration of recovery. Muscle wet weights (A), fiber CSAs (B), and anti‐laminin immunofluorescence staining of muscle sections (C). Data are presented as the mean + SE. *P < 0.05 versus control in each group, ^†^ *P* < 0.05 versus ipsilateral muscle in 72‐h recovery group, ^‡^ *P* < 0.05 versus ipsilateral muscle in 24‐h recovery group.

We first investigated the influence of shortening the recovery period on mechanical parameters including maximal isometric torque and impulses. The repetition of resistance exercise with 72 and 24 h of recovery led to gradual increases in maximal isometric torque, with a significant difference after 18 sessions. By contrast, repetition of resistance exercise with 8 h of recovery notably decreased the maximal isometric torque by the fourth session, after which the torque plateaued (Fig. 1A and B). The total number of impulses in each session was not markedly changed by the repetition of resistance exercise with 72‐h and 24‐h recovery periods. However, repetition of resistance exercise with an 8‐h recovery period markedly decreased the total number of impulses in each session by the fourth session, after which the value again plateaued again (Fig. 1C and D). We next investigated the muscle‐hypertrophic effects of each treatment. Repetition of resistance exercise with 72 and 24 h of recovery increased muscle wet weight and fiber CSA, but that with 8 h of recovery did not (Fig. 2A–C). These results indicate that excessive shortening of the recovery period disrupted the muscle‐hypertrophic effect of multiple sessions of resistance exercise.

### Muscle protein degradation systems

**Figure 3:**
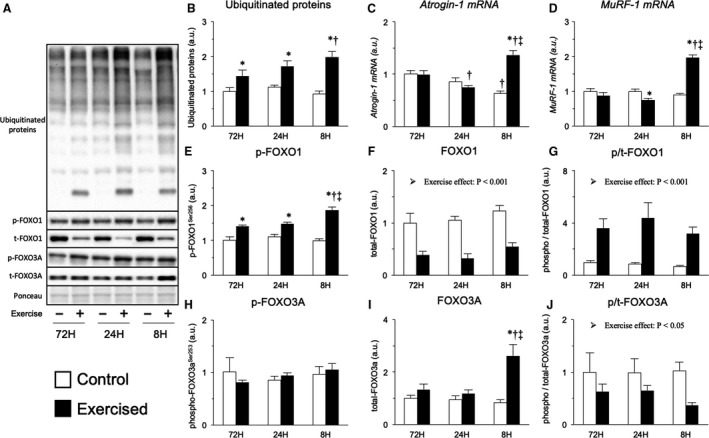
Protein expression, mRNA expression, and protein phosphorylation involved in the ubiquitin‐proteasome system after bouts of resistance exercise in rat skeletal muscle. Representative bands (A), ubiquitinated protein expression (B), expression of genes encoding atrogin‐1 and MuRF‐1 (C and D), phosphorylated and total protein expression and ratios of FOXO1 (E–G) and FOXO3A (H–J). Data are expressed relative to the no exercise 72‐h group and presented as the mean + SE. *P < 0.05 versus control in each group, ^†^ *P* < 0.05 versus ipsilateral muscle in 72‐h recovery group, ^‡^ *P* < 0.05 versus ipsilateral muscle in 24‐h recovery group.

To determine the contributions of the protein degradation systems to the lack of a muscle‐hypertrophic effect in the 8‐h recovery group, we first measured the factors involved in the ubiquitin‐proteasome system. Protein ubiquitination was observed in all groups, the magnitude of which was increased with shortening of the recovery period (Fig. 3B). The expression levels of the genes encoding atrogin‐1 and Muscle RING‐finger protein‐1 (MuRF‐1), which are E3 ubiquitin ligases involved in protein ubiquitination, were slightly decreased upon repetition of exercise with a 24‐h recovery period but were highly increased upon repetition of exercise with an 8‐h recovery period (Fig. 3C and 3). The transcription of E3 ubiquitin ligases during major protein degradation states (e.g., disuse, denervation) is mediated by the dephosphorylation and nuclear translocation of FOXO (Sandri et al. 2004). Therefore, we next measured the phosphorylation of FOXO1 and FOXO3A. However, the phosphorylation ratio of FOXO1 was markedly increased by exercise, but no significant difference was observed among the groups (main effect of exercise, Fig. 3E–G). Moreover, while the total expression of FOXO3A was increased only in the 8‐h recovery group, the phosphorylation of FOXO3A was decreased by exercise (main effect of exercise, Fig. 3H–J). We additionally assessed the translocation of FOXO proteins, but no obvious changes (interaction of exercise and recovery) were observed (Fig. S1 [10.6084/m9.figshare.7634840]). Although the upstream transcription factor responsible for these effects has not been identified, these results indicate that the repetition of resistance exercise with an excessively shortened recovery period accelerates protein ubiquitination.

**Figure 4:**
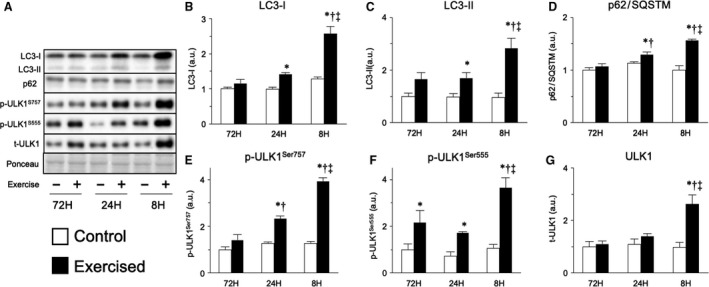
Protein expression involved in autophagy‐lysosome system after bouts of resistance exercise in rat skeletal muscle. Representative bands (A), protein expression of LC3‐I (B), LC3‐II (C), p62/SQSTM (D), phosphorylated ULK1 (Ser757, E), phosphorylated ULK1 (Ser555, F), and ULK1 (G). Data are expressed relative to the no exercise 72‐h group and presented as the mean + SE. *P < 0.05 versus control in each group, ^†^ *P* < 0.05 versus ipsilateral muscle in 72‐h group, ^‡^ *P* < 0.05 versus ipsilateral muscle in 24‐h group.

We next measured the factors involved in autophagy, the other major protein degradation system. LC3‐II is the phosphatidylethanolamine‐conjugated form of LC3‐I, which attaches to autophagosome membranes and is widely used as a marker of autophagosome formation. Bouts of resistance exercise increased LC3‐II expression, and the shortening of the recovery period heightened this effect (Fig. 4C). A similar result was observed for p62, a cargo receptor for ubiquitinated proteins that are degraded by autophagy (Fig. 4D). ULK1 is a factor that controls the initiation of isolation membrane formation and plays different roles depending on which of its amino acid sites are phosphorylated. Phosphorylated ULK1^Ser757^ exerts an inhibitory effect on autophagosome formation by downregulating the membrane elongation step; this form of ULK1 is primarily phosphorylated by mammalian/mechanistic target of rapamycin complex 1 (mTORC1, Chan, 2009). In contrast, phosphorylated ULK1^Ser555^, which is primarily phosphorylated by AMPK (Mao and Klionsky, 2011), facilitates autophagosome formation by upregulating the membrane elongation step. We found that bouts of resistance exercise with shorter recovery periods (24 and 8 h) increased the expression of phosphorylated ULK1^Ser757^, with the degree of expression increasing as the recovery period became shorter (Fig. 4E). However, bouts of resistance exercise also increased the expression of phosphorylated ULK^Ser555^ in all groups, especially in the 8‐h recovery group (Fig. 4F). These observations indicate that both phosphorylation sites in ULK1 (Ser757 and Ser555) were phosphorylated and that the repetition of resistance exercise with shorter recovery periods accelerated autophagosome formation.

### mTORC1 signaling

**Figure 5:**
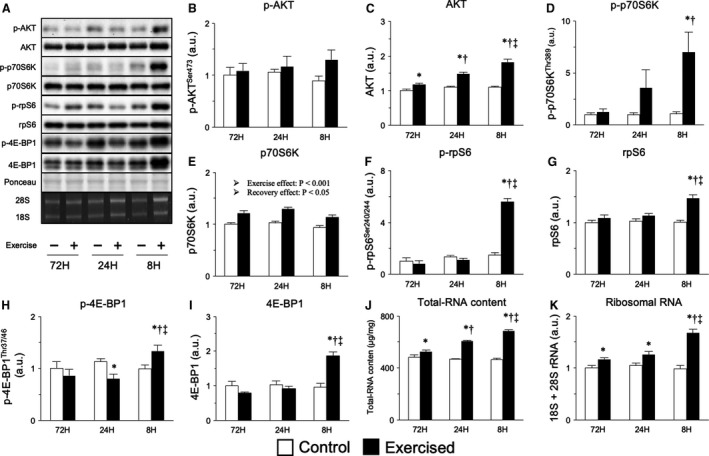
Protein expression involved in mTORC1 signaling after bouts of resistance exercise in rat skeletal muscle. Representative bands (A), protein expression of phosphorylated AKT (Ser473, B), AKT (C), phosphorylated p70S6K (Thr389, D), p70S6K (E), phosphorylated rpS6 (Ser240/244, F), rpS6 (G), phosphorylated 4E‐BP1 (Thr37/46, H), 4E‐BP1 (I), total RNA content (J), and ribosomal RNA expression (K) per tissue. Data are expressed relative to the no exercise 72‐h group and presented as the mean + SE. *P < 0.05 versus control in each group, ^†^ *P* < 0.05 versus ipsilateral muscle in 72‐h group, ^‡^ *P* < 0.05 versus ipsilateral muscle in 24‐h group.

mTORC1 signaling is a primary regulator of muscle protein synthesis and hypertrophy in resistance exercise and also acts as an inhibitor of the protein degradation systems. We first investigated AKT, an effector of mTORC1 signaling that acts as a countermeasure for the ubiquitin‐proteasome system via the phosphorylation of FOXO (Sandri et al., 2004). As shown in Figure 5, bouts of resistance exercise increased the expression of AKT in all groups, with greater increases in the groups with shorter recovery periods; in contrast, no significant difference was observed in the phosphorylated form of AKT (Fig. 5B and 5). However, bouts of resistance exercise with an 8‐h recovery period resulted in the phosphorylation of p70S6K, a downstream target of mTORC1 and an indicator of mTORC1 signaling activity (Fig. 5D). Moreover, bouts of resistance exercise increased the level of total p70S6K (main effect of exercise) with an 8‐h recovery period leading to only a slight increase (main effect of recovery, Fig. 5E). Additionally, levels of phosphorylated and total rpS6, a downstream target of p70S6K, were increased only by bouts of resistance exercise with an 8‐h recovery period (Fig. 5F and G). Similar results were observed for 4E‐BP1, another downstream target of mTORC1 (Fig. 5H and I). We additionally investigated ribosomal biogenesis, which is partially regulated by mTORC1. Bouts of resistance exercise increased total RNA and ribosomal RNA contents, with shorter recovery periods strengthening this effect (Fig. 5J and K). These results suggest that bouts of resistance exercise activate the mTORC1 signaling pathway, especially those with an excessively short (8 h) recovery period.

### Mitochondrial content and dynamics

**Figure 6:**
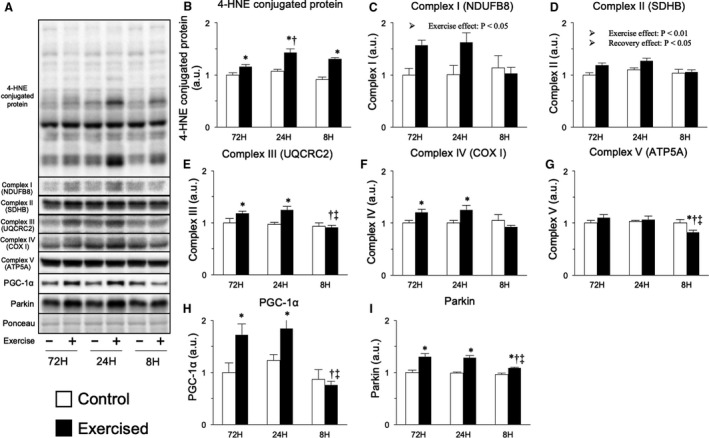
Protein expression related to mitochondrial content. Representative bands (A), protein expression of 4‐HNE‐conjugated protein (B), OXPHOS (C–G), PGC‐1*α* (H), and parkin (I). Data are expressed relative to the no exercise 72‐h group and presented as the mean + SE. *P < 0.05 versus control in each group, ^†^ *P* < 0.05 versus ipsilateral muscle in 72‐h group, ^‡^ *P* < 0.05 versus ipsilateral muscle in 24‐h group.

To evaluate the influence of mitochondrial adaptation on the protein degradation systems in relation to the shortening of the recovery period, we next investigated mitochondrial contents and dynamics. First, we investigated oxidative stress, which exerts toxic effects on mitochondria and is reported to increase following exhaustive resistance exercise (14, 43). Levels of 4‐HNE‐conjugated proteins, markers of oxidative stress, were increased by bouts of resistance exercise, and the shortening of the recovery period heightened this effect; however, excessive shortening of the recovery period attenuated 4‐HNE‐conjugated protein levels to the same level observed in the general recovery group (Fig. 6B). We next investigated OXPHOS proteins, which are indicators of mitochondrial content. Levels of complex I and II proteins were increased by resistance exercise (main effect of exercise), and levels of complex II proteins were slightly reduced by an excessively shortened recovery period (main effect of recovery, Fig. 6C and 6). Bouts of resistance exercise with 72 and 24 h of recovery increased the levels of complex III and IV proteins, but resistance exercise with 8 h of recovery did not (Fig. 6E and 6). Additionally, bouts of resistance exercise with 72 and 24 h of recovery did not change protein levels of complex V, while those with 8 h of recovery decreased complex V protein levels (Fig. 6G). We next investigated factors involved in mitochondrial biogenesis and breakdown. The expression level of PGC‐1*α*, a master regulator of mitochondrial biogenesis, was increased by bouts of resistance exercise with 72 and 24 h of recovery but not by those with 8 h of recovery (Fig. 6H). In contrast, bouts of resistance exercise increased the expression level of parkin, a mediator of mitochondrial autophagy, in all groups, though the degree of upregulation was lower in the 8‐h recovery group (Fig. 6I). These results suggest that excessive shortening of the recovery period between sessions inhibited exercise‐induced increases in mitochondrial content.

**Figure 7:**
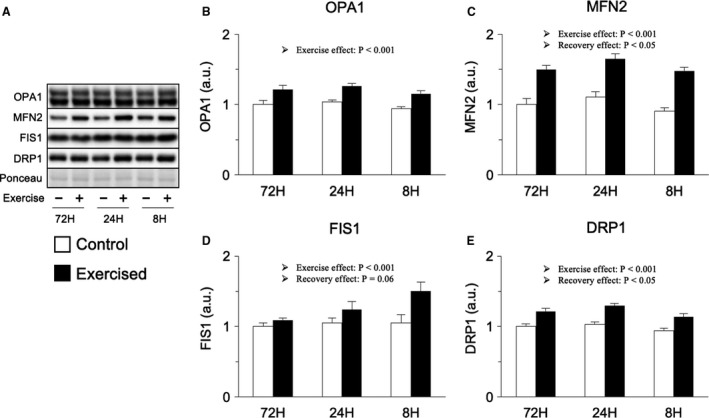
Protein expression involved in mitochondrial dynamics. Representative bands (A), protein expression of OPA1 (B), MFN2 (C), FIS1 (D), and DRP1 (F). Data are expressed relative to the no exercise 72‐h group and presented as the mean + SE. *P < 0.05 versus control in each group, ^†^ *P* < 0.05 versus ipsilateral muscle in 72‐h group, ^‡^ *P* < 0.05 versus ipsilateral muscle in 24‐h group.

Next, we investigated proteins involved in regulating mitochondrial dynamics. Bouts of resistance exercise increased the expression levels of mitochondrial fusion‐related proteins, such as OPA1 and MFN2 (main effect of exercise), but shortening the recovery period did not result in noticeable effects (Fig. 7B and C). In contrast, bouts of resistance exercise increased the expression levels of mitochondrial fission‐related proteins, such as FIS1 and DRP1 (main effect of exercise), with slightly higher increases in the shorter recovery groups (main effect of recovery), although no interaction between exercise and recovery was observed (Fig. 7D and E). Therefore, mitochondrial dynamics were not markedly changed by the shortening of the recovery period between sessions.

### AMPK and inflammatory response

**Figure 8:**
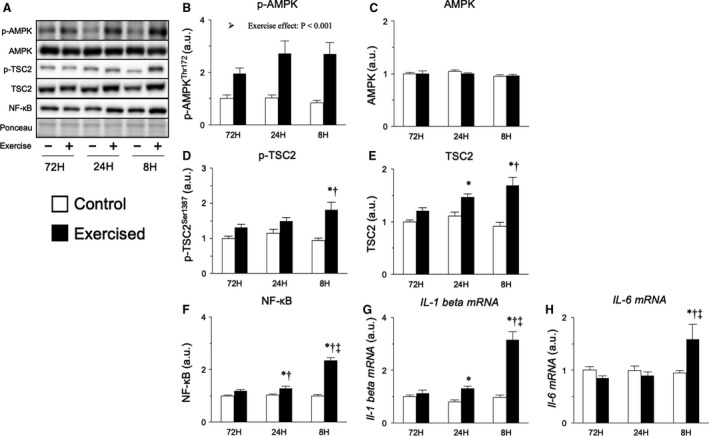
Protein and mRNA expression related to AMPK and inflammatory response. Representative bands (A), protein expression of phosphorylated AMPK (Thr172, B), AMPK (C), phosphorylated TSC2 (Ser1387, D), TSC2 (E), NF‐*κ*B (F), expression of genes encoding IL‐1*β* (G), and IL‐6 (H). Data are expressed relative to the no exercise 72‐h group and presented as the mean + SE. *P < 0.05 versus control in each group, ^†^ *P* < 0.05 versus ipsilateral muscle in 72‐h group, ^‡^ *P* < 0.05 versus ipsilateral muscle in 24‐h group.

Finally, we attempted to explore effects on AMPK and inflammatory responses, which are known to be activated by the repetition of resistance exercise with short recovery periods, accelerating the protein degradation systems (Coffey et al., 2007; Takegaki et al., 2017). Bouts of resistance exercise increased the expression level of phosphorylated AMPK (main effect of exercise), but no interaction of exercise and recovery was observed (Fig. 8B). The expression level of phosphorylated TSC2, a downstream target of AMPK, was increased only by bouts of resistance exercise with 8 h of recovery (Fig. 8D). We next measured inflammatory signals, which also activate muscle catabolic signals and inhibit protein synthesis (Cai et al., 2004; Li et al., 2009; Hardee et al., 2017). The expression level of NF‐*κ*B, a mediator of the inflammatory response, was increased by bouts of resistance exercise with shorter recovery periods (24 and 8 h), with the 8‐h recovery group showing the strongest effect (Fig. 8F). Additionally, NF‐*κ*B was translocated into the nucleus in the shorter recovery groups to a greater extent (Fig. S1 [10.6084/m9.figshare.7634840]). Similar results were observed when investigating the expression of the gene encoding IL‐1*β* (Fig. 8G). In contrast, the mRNA expression of *Il6* was increased only by bouts of resistance exercise with 8 h of recovery (Fig. 8H). These observations indicate that bouts of resistance exercise, especially those with excessively short recovery periods, induce the activation of an inflammatory response.

## Discussion

In the present study, we investigated the effects of reducing the recovery between bouts of resistance exercise by varying the duration of the resting interval between 18 successive bouts of resistance exercise and observing the corresponding changes in muscle hypertrophy. The main findings of this study are as follows: (1) 18 sessions of resistance exercise with 72‐h and 24‐h recovery periods increased the maximal isometric torque, while the same exercise regimen with an 8‐h recovery period decreased maximal isometric torque; (2) 18 sessions of resistance exercise with 72‐h and 24‐h recovery periods, but not with an 8‐h recovery period, induced muscle hypertrophy; (3) 18 sessions of resistance exercise with an 8‐h recovery period strongly evoked protein ubiquitination and autophagosome formation; and (4) rats subjected to 8‐h recovery periods between bouts exhibited inflammatory responses. These results suggest that excessive shortening of recovery periods between exercise bouts decreases muscle‐hypertrophic effects, which may be due to the upregulation of protein degradation systems, enhancing inflammatory responses.

In this study, excessive shortening of the recovery period caused an immediate decrease in the maximal isometric torque and impulses. A previous study showed that 10 sets of 10 maximal concentric knee extensions reduced maximal isometric torque to 60% of the maximum value immediately after the exercise, but that the maximal torque was restored within 24 h in humans (Michaut et al., 2003). Gibala et al. reported similar results (Gibala et al., 1995), suggesting that 8‐h intervals were insufficient for recovery in the present study. These facts also suggest that the repetition of resistance exercise at intervals that are short but enough to recover maximal torque may accelerate the increase in maximal torque.

Although the influence of shortening of the recovery period on muscle anabolic and catabolic responses has been partially reported (Coffey et al., 2007; Takegaki et al., 2017), the detailed influence on the muscle‐hypertrophic effect has not been reported. To our knowledge, therefore, our study is the first to demonstrate that excessive shortening of the recovery period blunts muscle‐hypertrophic effect induced by bouts of resistance exercise. To determine the mechanism of this inhibition of muscle hypertrophy, we focused on the protein degradation systems. Consistent with the study reporting activation of the ubiquitin‐proteasome system (Coffey et al., 2007), we demonstrated that not only the ubiquitin‐proteasome system but also the autophagy‐lysosome system was activated by shortening of the recovery period. Thus, excessive shortening of the recovery period activates both protein degradation systems, which likely blunts muscle hypertrophy induced by bouts of resistance exercise.

FOXO proteins are transcription factors of ubiquitin ligases as well as autophagy controllers (Sandri et al., 2004; Stitt et al., 2004; Mammucari et al., 2007). Interestingly, although the phosphorylation status and nuclear expression level of FOXO3A were not changed, its total expression level was markedly increased in the 8‐h recovery group. The mechanism for the increase in FOXO3A expression is currently unclear. However, under catabolic states such as immobilization, FOXO3A expression is known to increase in skeletal muscle (Okamoto and Machida, 2017; Kawanishi et al., 2018). Considering these facts, FOXO3A is possibly involved in the bluntation of the muscle‐hypertrophic effect observed in the group with excessive shortening of the recovery period; thus, the involvement of FOXO proteins in the acceleration of protein ubiquitination, ubiquitin ligase mRNA expression, and autophagosome formation should be clarified in future investigations.

We next explored mTORC1 signaling, which acts as a countermeasure to protein degradation systems and activates protein synthesis (Bodine et al., 2001a, 2001b; Stitt et al., 2004; Drummond et al., 2009). In the present study, we did not observe changes in the phosphorylation of AKT, which counteracts the activity of FOXO proteins (Sandri et al., 2004; Stitt et al., 2004). In contrast, the repetition of resistance exercise with excessive shortening of the recovery period activated mTORC1 signaling, with effects observed even at 48 h after the final exercise session. In this study, although protein synthesis in each muscle was not measured, the ribosome content increased in part due to mTORC1, indicating that protein synthesis was strongly activated in the 8‐h recovery group. Therefore, at least, suppression of the muscle‐hypertrophic effect in the group with excessive shortening of the recovery period would not be characterized by inactivation of mTORC1 signaling.

We also investigated mitochondrial adaptations as a potential means of regulating protein degradation systems. As mentioned above, we recently demonstrated that the repetition of resistance exercise with excessively shortened recovery periods results in acute induction of oxidative stress, an inducer of mitochondrial toxicity (Kitaoka et al., 2016b; Takegaki et al., 2017). However, unexpectedly, 18 sessions of resistance exercise with excessively short recovery periods attenuated the increase in oxidative stress and only slightly reduced the mitochondrial content in the present study. Intracellular oxidative stress status is sensitively controlled by the balance of oxidant and antioxidant levels (Kregel and Zhang 2007), and the 18 resistance exercise sessions undertaken in this study might have altered this balance, reducing oxidative stress levels. Moreover, no obvious changes were observed in mitochondrial dynamic‐related factors in the present study. Therefore, while the finding that mitochondrial adaptation was partially inhibited by excessive shortening of the recovery period is meaningful, it is likely that mitochondrial adaptation is not responsible for the upregulation of protein degradation signals induced by shortened recovery in the present study.

To explore other mechanisms potentially responsible for the upregulation of protein degradation, we analyzed AMPK and inflammatory signals. However, although prior studies have shown that high‐frequency resistance training induces acute phosphorylation of AMPK at high levels that activates protein degradation systems (Coffey et al., 2007; Nakashima and Yakabe, 2007; Mao and Klionsky, 2011; Takegaki et al., 2017), we failed to detect differences between the exercised groups after 18 sessions of resistance exercise. In our current study, rats were fasted overnight before dissection, which should lead to low energy status. Therefore, the overnight fast and increased energy consumption caused by improvements in basal metabolism may synergistically induce a low energy state in exercised muscle, resulting in the phosphorylation of AMPK. In contrast, the phosphorylation of TSC2, a downstream target of AMPK, was upregulated in groups with shorter recovery periods in the present study. Therefore, at a time point earlier than 48 h, AMPK should be phosphorylated in recovery period‐dependent manner, contributing to the activation of protein degradation systems. Furthermore, we found that the repetition of resistance exercise with shorter recovery periods upregulated inflammatory signals to a greater extent in the present study. NF‐*κ*B is widely recognized as an inflammatory mediator and known to activate the transcription of inflammatory cytokines in the nucleus (Liu and Malik 2006). Previous studies have demonstrated that inflammatory cytokines such as TNF‐*α*, IL‐1*β*, and IL‐6 activate protein degradation systems and promote muscle atrophy in vivo and in vitro (Bodine et al., 2001a, 2001b; Cai et al., 2004; Janssen et al., 2005; Doyle et al., 2011). Although the detailed mechanisms have not been clarified, there are some previous in vitro studies showing that p38 mitogen‐activated protein kinase (MAPK) or FOXO4 are involved in the activation of protein degradation systems by inflammatory cytokines (Li et al., 2005; Moylan et al., 2008; Doyle et al., 2011), and NF‐*κ*B activation itself is known to promote the transcription of the MuRF‐1‐encoding gene and cause skeletal muscle atrophy in vivo (Cai et al., 2004). Additionally, Coffey et al. reported that single and multiple bouts of resistance exercise induced the phosphorylation of I*κ*B kinase *β* (IKK*β*), an activator of NF‐*κ*B, at least 3 h after the last exercise bout in skeletal muscle (Coffey et al., 2007). These facts led us to consider that repetition of resistance exercise with an excessively short recovery period kept NF‐*κ*B in the activated state throughout the training period, leading to inflammation upregulation in the exercised muscle and resulting in muscle hypertrophy attenuation. To determine the contribution of inflammatory signals to the attenuation of muscle hypertrophy, future studies should examine whether inhibiting the activation of NF‐*κ*B counteracts the attenuation of the resistance training‐induced muscle hypertrophy caused by excessive shortening of the recovery period.

In conclusion, the repetition of resistance exercise with an excessively shortened recovery period upregulated protein degradation systems and reduced muscle strengthening and hypertrophic effects. The upregulation of protein degradation signals was likely caused by local inflammation in the skeletal muscle. A potential weakness of the study is that we did not evaluate muscle injury. Since we used an isometric contraction model for the resistance exercise, muscle injury is less likely to occur. However, activation of mTORC1 signaling and inflammation are also characteristics of muscle injury (Baumann et al., 2016; Baumert et al., 2016), and thus, muscle injury might suitably explain the changes observed in the present study. Therefore, further studies evaluating muscle injury are required to elucidate the detailed mechanisms involved in the attenuation of resistance exercise‐induced muscle hypertrophy caused by excessive shortening of the recovery period. Finally, in the present study, we did not match the total intervention periods among the groups, and thus, the groups with shorter recovery periods completed their 18 sessions earlier. These facts indicate the necessity for further studies matching the intervention periods to elucidate additional changes in exercise‐induced effects following a shortened recovery period; however, the most important fact is that repetition of resistance exercise with a moderately shortened recovery period induces muscle strengthening and hypertrophic effects earlier than a normal recovery period. Therefore, consideration of the optimal period of recovery may enable the development of the most time‐effective protocols for resistance training.

## Conflict of Interest

The authors declare no conflicts of interest.

## Supporting information




**Figure S1.** Nuclear translocation of FOXO1, FOXO3A, and NF‐*κ*B. Representative bands (A), cytoplasmic FOXO1 (B), nuclear FOXO1 (C), cytoplasmic FOXO3A (D), nuclear FOXO3A (E), cytoplasmic NF‐*κ*B (F), nuclear NF‐*κ*B (G). Data are expressed relative to the no exercise 72‐h group and presented as the mean + SE. *P < 0.05 versus control in each group, ^†^ *P* < 0.05 versus ipsilateral muscle in 72‐h group, ^‡^ *P* < 0.05 versus ipsilateral muscle in 24‐h group.
