# Supplementary figures and images for: Influence of shortened recovery between resistance exercise sessions on muscle‐hypertrophic effect in rat skeletal muscle
Source: Physiol Rep. 2019 Jun 28;7(13):e14155. doi: 10.14814/phy2.14155 (PMC6598394; doi:10.14814/phy2.14155)

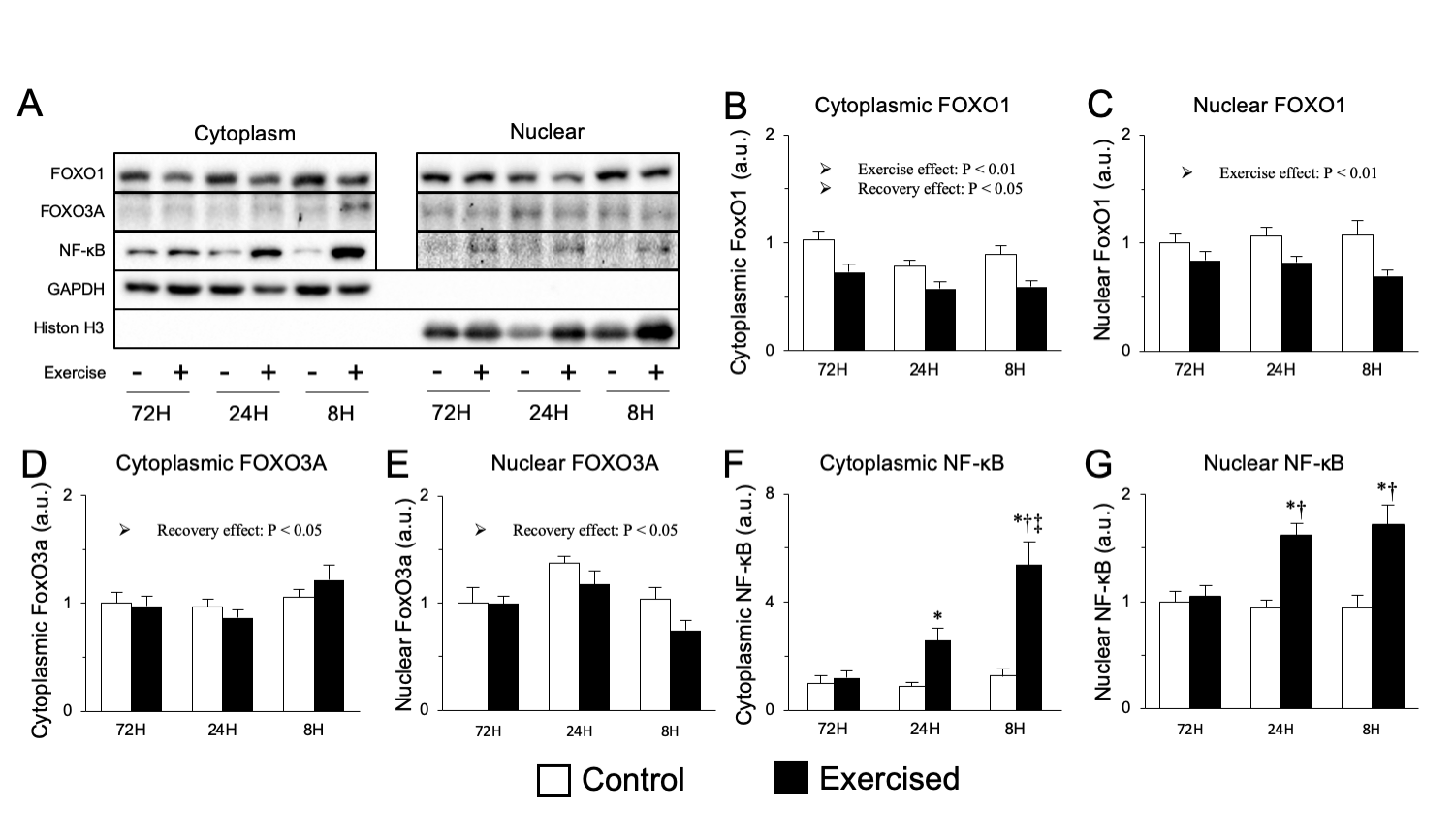

Supplement: Supplementary file 1 — Figure S1. Nuclear translocation of FOXO1, FOXO3A, and NF‐κB. Representative bands (A), cytoplasmic FOXO1 (B), nuclear FOXO1 (C), cytoplasmic FOXO3A (D), nuclear FOXO3A (E), cytoplasmic NF‐κB (F), nuclear NF‐κB (G). Data are expressed relative to the no exercise 72‐h group and presented as the mean + SE. *P < 0.05 versus control in each group, † P < 0.05 versus ipsilateral muscle in 72‐h group, ‡ P < 0.05 versus ipsilateral muscle in 24‐h group. [file PHY2-7-e14155-s001.tiff]
